# Binding of *Escherichia coli* Does Not Protect Tulane Virus from Heat-Inactivation Regardless the Expression of HBGA-Like Molecules

**DOI:** 10.3389/fmicb.2017.01746

**Published:** 2017-09-21

**Authors:** Qianqian Li, Dapeng Wang, David Yang, Lei Shan, Peng Tian

**Affiliations:** ^1^Department of Bioengineering, Shanghai Institute of Technology Shanghai, China; ^2^Produce Safety and Microbiology Research Unit, Western Regional Research Center, Agricultural Research Service, United States Department of Agriculture, Albany CA, United States; ^3^MOST-USDA Joint Research Center for Food Safety, School of Agriculture and Biology, Shanghai Jiao Tong University Shanghai, China

**Keywords:** norovirus, histo-blood group antigens, Tulane virus, *Escherichia coli*, heat-denaturation, protection, ELISA, RT-qPCR

## Abstract

Histo-blood group antigens (HBGAs) are considered as receptors/co-receptors for human norovirus (HuNoV). It has been reported that binding of HuNoV-derived virus-like particles (VLPs) to HBGA-like molecules-expressing bacteria increased the stability of VLPs to heat-denaturation (HD). In this study, we tested for HBGA-like-binding-conveyed protection against HD on viral replication using Tulane virus (TV) and *Escherichia coli* O86:H2 (O86:H2), with *E. coli* K-12 (K-12) used as a control. Expression of HBGA type B was confirmed by ELISA in O86:H2 but not in K-12. Binding of TV was confirmed by ELISA in O86:H2 (P/N = 2.23) but not in K-12 (P/N = 1.90). Pre-incubation of TV with free HBGA could completely inhibit its ability to bind to O86:H2 (*p* = 0.004), while producing no significant change in its ability to bind K-12 (*p* = 0.635). We utilized a bacterial-capture-RT-qPCR procedure to confirm that both bacterial strains were capable of binding TV, and that O86:H2 exhibited fivefold greater binding capacity than K-12. Pre-incubation of TV with free HBGA would partially inhibit the binding of TV to O86:H2 (*p* = 0.047). In contrast, not only did pre-incubation of TV with free HBGA not inhibit the binding of TV to K-12, binding was slightly enhanced (*p* = 0.13). The viral infectivity assay allowed us to conduct a direct evaluation of the ability of HBGA-like-bound bacteria to confer HD protection to TV. Prior to inoculate to LLC-MK2 cells, TV was incubated with each bacterial strain at ratios of 1:0, 1:1 and 100:1, then both partially and fully HD. The viral amplification was quantitated by RT-qPCR 48 h later. The binding of bacteria to TV reduced viral replication in a dose-dependent matter. We found that neither bound O86:H2 nor K-12 conferred protection of TV against partial or full HD conditions. Partial HD reduction of viral replication was not significantly impacted by the binding of either bacterial strain, with infectivity losses of 99.03, 99.42, 96.32, 96.10, and 98.88% for TV w/o bacteria, TV w/O86:H2 (1:1), TV w/O86:H2 (100:1), TV w/K-12 (1:1), and TV w/K-12 (100:1), respectively. Full HD reduction of viral replication was not impacted by the binding of either bacterial strain, as full loss of infectivity was observed in all cases.

## Introduction

Human noroviruses (HuNoVs) are the major cause of outbreaks of acute non-bacterial gastroenteritis. Noroviruses have a single-stranded, positive-sense RNA genome which contains three open reading frames (ORFs) encoding non-structural proteins (ORF1), a capsid protein VP1 (ORF2), and a minor capsid protein VP2 (ORF3) (Xi et al., 1990; Jiang et al., 1993; Hardy, 2005). HuNoVs are difficult to grow in cell culture, and culturable viruses such as Tulane virus (TV), murine norovirus (MNV), and feline calicivirus (FCV) are often utilized as surrogates for studying the fundamental biology of HuNoV, such as viral replication pattern, and mechanism of infection (Cannon et al., 2006; Wobus et al., 2006; Farkas et al., 2008; Tian et al., 2013).

The relationship between histo-blood group antigens (HBGAs) and susceptibility to HuNoV infections was noticed by several research groups (Parrino et al., 1977; Gary et al., 1987). HBGAs can be either lipid-linked or protein-linked, and can present as either membrane-associated or secreted. HuNoVs were found to interact with cell-surface-displayed HBGAs, and were thought to be important for viral infection (Harrington et al., 2002; Huang et al., 2005). Although most HuNoVs preferentially bind to one-or-more HBGAs, some norovirus genotypes do not bind or poorly bind HBGAs (Tan and Jiang, 2005). TV binds to type-A and type-B HBGAs (Farkas et al., 2010). Surrogate viruses also bind to molecules other than HBGAs. MNV-1 and FCV bind to sialic acid (Stuart and Brown, 2007; Taube et al., 2009).

An early study demonstrated that enteric bacteria can enhance poliovirus replication *in vivo* and in culture (Kuss et al., 2011). It has been hypothesized that bacterial components might increase viral receptor binding or viral shedding. HBGA-like moieties have been reported to be present on the surface of some enteric bacteria (Springer et al., 1961; Yi et al., 2006; Miura et al., 2013; Li et al., 2015). Interactions between bacterial-expressed HBGA-like molecules and HuNoV/surrogates and their role in viral replication and resistance/survival have been investigated recently. HuNoV was found to bind specifically to the type H HBGA that could be found on *Enterobacter cloacae* (Miura et al., 2013). Li et al. (2015) reported that HuNoV viral-like particles (VLPs) bound to HBGA-like molecules-expressing bacteria. Almand et al. (2017) recently reported that HuNoV and TV were able to bind to both Gram-positive and -negative bacteria selected from human gut microbiota, with TEM showing that the viruses could be found bound to the bacterial outer cell membrane, intestinal pili, or both. The interaction between HuNoV/surrogates and bacteria seemed specific as Turnip Crinkle virus, a plant virus that closely resembles HuNoV and was used as a control, did not bind to these bacteria (Almand et al., 2017). However, there was no evidence that these tested bacteria all expressed HBGA-like molecules, and therefore remains unclear if bacterial molecules other than HBGA are involved in the binding of the viruses.

The exact role HBGA-like-expressing bacteria play in the life-cycle of enteric viruses and norovirus remains unknown. Jones et al. (2014) demonstrated that the infection of B cells by MNV was promoted by *Enterobacter cloacae* expressing type-H HBGA-like molecules or synthetic HBGA. Karst and Wobus (2015) proposed that HBGA-like molecules-expressing bacteria might help HuNoV and MNV to transcytose across intestinal epithelial cells. Robinson et al. (2014) reported that the environmental stability and target cell attachment of poliovirus was enhanced by the binding of bacterial surface polysaccharides. Li et al. (2015) recently reported that the binding of HBGA-like molecules-expressing bacteria to HuNoV VLPs increased the ability of the viral capsids to resist heat denaturation. Although the study suggests that the binding of HBGA-like molecules-expressing bacteria protected HuNoV VLPs from heat denaturation, it does not extend to drawing conclusions concerning the potential for HBGA-like molecules-expressing bacteria to protect HuNoV from heat-denaturation-related reduction of infectivity. In this study, we used TV as a surrogate to test the impact of HBGA-like molecules-expressing bacteria on viral infectivity after heat treatment.

Tulane virus is a calicivirus isolated from stools of rhesus macaques at the Tulane National Primate Research Center (Farkas et al., 2008). TV replicates *in vitro* in rhesus monkey kidney immortal cell culture (LLC-MK2) and causes typical cytopathic effect (CPE). TV recognizes both type-A and type-B HBGAs and receptors for infection, preferring the latter (Farkas et al., 2010). The virus could be partially inactivated at 50°C for 10 min. and completely inactivated at 56°C for 30 min (Tian et al., 2013). For this study, *E. coli* O86:H2 was selected for its ability to express type-B HBGA-like molecules (Yi et al., 2006). The viral replication was measured by a newly developed cell-culture-mediated amplification RT-qPCR assay (Xu et al., 2015).

## Materials and Methods

### Sources and Preparation of Bacteria, Virus, Saliva, and Reagents

Tulane virus (TV) was kindly provided by Dr. Jiang (Division of Infectious Diseases, Cincinnati Children’s Hospital Medical Center, Cincinnati, OH, United States), and viral cultured in LLC-MK2 (American Type Culture Collection, Manassas, VA, United States) cell culture (Tian et al., 2013). *E. coli* O86:H2 and K-12 were kindly provided by Dr. Wang (Peng G. Wang, Department of Chemistry, Georgia State University, Atlanta, GA, United States). Bacterial strains were cultured in 5 mL aliquots of LB medium at 37°C, at moderate mixing (200 rpm) for overnight. The cultured bacteria were pelleted by centrifugation (8,000 RCF for 2 min), and the bacterial pellet was washed three times by vortex re-suspension with PBS and re-pelleted by centrifugation to produce working stocks of media-free cell suspensions.

Human saliva was sourced from three blood-type B volunteers under approval by the Institutional and Location Bio-safety Committees (IBC and LBC) of Shanghai Jiao Tong University, Institutional Ethics Committees (IEC) of College of Agriculture and Biology, Shanghai Jiao Tong University, and written informed consent was obtained from the volunteers. With no personal information collected, about 2 ml saliva was collected from each volunteer, immediately aggregated, then mixed. The mixed saliva was boiled for 5 min, then centrifuged at 10,000 ×*g* for 5 min. The clarified supernatant was aliquotted and stored at -20°C (Wang et al., 2017).

Monoclonal antibodies (MAbs) against blood group antigen-precursor “H” (“BG1”), type A (“BG2”), type B (“BG3”), H1 (“BG4”), Lewis a (“BG5”), Lewis b (“BG6”), Lewis x (“BG7”), and Lewis y (“BG8”) were purchased from a commercial source (BioLegend, Inc., San Diego, CA, United States). AP-conjugated goat anti-mouse IgM and IgG; and AP-conjugated goat anti-rabbit IgG were purchased from a commercial source (ZYMED Laboratories, South San Francisco, CA, United States). Rabbit-anti-TV sera was kindly provided by Dr. Jiang (Division of Infectious Diseases, Cincinnati Children’s Hospital Medical Center, Cincinnati, OH, United States). Type III porcine gastric mucin (PGM) was purchased from a commercial source (Sigma, St. Louis, MI, United States).

### Detection of HBGA Present in Bacteria by ELISA

Both *E. coli* O86:H2 and K-12 media-free bacterial suspensions were adjusted with PBS to an OD_600_ of 0.1. Each OD_600_ 0.1 bacterial suspension was seeded into individual wells of immunoassay strips (Nunc Immuno Modules, Thermo, MA, United States). Well were air-dried. Each well was subsequently rinsed three times with PBS, then air-dried. To block unbound well surfaces, 200 μl of 10% skim milk (Difco Laboratories, Detroit, MI, United States) in PBS was added to each well and incubated at 37°C for 2 h, then carefully removed and rinsed once with PBS. The commercial stocks of the MAbs were diluted 1:50 in PBS and added into each well and incubated at 37°C for 1 h. Unbound MAbs were removed by washing wells three times with 250 μl of TBS containing 0.5% Tween-20 (TBST). One hundred μl of goat anti-mouse IgG (MAbs BG2, 4, and 5) or goat anti-mouse IgM (MAbs BG1, 3, 6, 7, and 8) conjugated to alkaline phosphatase (AP) and diluted 1:3000 in TBST were added to appropriate wells, and the wells were incubated at 37°C for 1 h, followed by three washes with 250 μl of TBST. The presence of well-bound bacteria-and-alkaline-phosphatase-conjugated-antibody complex was developed for detection by the addition of 100 μl of alkaline phosphatase substrate [*p*-nitrophenyl phosphate, disodium salt substrate at a concentration of 1.0 mg/ml in diethanolamine substrate buffer (Pierce, Rockford, IL, United States)] to each well. The color reaction is quantitated using a spectrophotometric plate reader (Spectramax ELISA reader, Molecular Devices, Sunnyvale, CA, United States) reading absorbance at 405 nm. *E. coli* K-12 was used as a control for non-specific binding. The P/N ratio was calculated using OD readings from wells omitted for primary antibodies as N.

### Detection of TV Binding to Bacteria by ELISA

To reduce the non-specific antibody binding of *E. coli*, Rabbit-anti TV antiserum was pre-absorbed with *E. coli* K12 extract. Briefly, *E. coli* K-12 media-free bacterial suspension was adjusted to OD_600_ of 0.1, and then lysed by ultrasonication (Qsonica Sonicator Q500, Fisher Scientific, United States) using 4-s bursts at 6-s intervals for 10 min (225 W) on ice. The cell lysate was clarified by centrifugation at 12,000 ×*g* at 4°C for 10 min, and the supernatant was collected. Rabbit-anti TV antiserum was diluted into the supernatant at a ratio of 1:100 and incubated at 37°C for 30 min. The supernatant w/antiserum was centrifuged at 12,000 ×*g* at 4°C for 10 min, and the supernatant was collected and stored at 4°C for further use.

Immunoassay plates were coated with bacteria and blocked with skim milk as described in the previous section. Free HBGA for receptor competition was made from mixing PGM (1.0 mg/ml) and type B saliva at a ratio of 10:1. Ninety microliters of TV stock were incubated with 10 μl of either free HBGA or PBS (as a control) and incubated at 37°C for 1 h. The TV stock w/free HBGA were then added to the coated-and-blocked wells, and incubated at 37°C for 1 h. Viral stock was carefully removed, and residual stock was removed from the wells by rinsing three times with PBS. Primary antibodies were introduced with the addition of 100 μl of diluted (1:3000 in PBS) rabbit-anti-TV antiserum to each well, incubation at 37°C for 1 h, then carefully removed. Residual, unbound primary antibodies were removed from the wells by rinsing three times with TBST. Secondary antibodies were introduced with the addition of 100 μl of diluted (1:10,000 in TBST) AP-conjugated goat anti-rabbit IgG to respective wells, and incubated at 37°C for 1 h. Residual, unbound secondary antibodies were removed from the wells by rinsing three times with TBST. The presence of well-bound bacteria-TV-and-alkaline-phosphatase-conjugated-antibody complex was developed as described in the previous section, as was the use of *E. coli* K-12 as a control for non-specific binding. The P/N ratio was calculated using OD readings from wells omitted for primary antibodies as N.

### Binding of TV to Bacteria Measured by RT-qPCR

Both *E. coli* O86:H2 and K-12 media-free bacterial suspensions were adjusted with PBS to approximately 10^8^ CFU/ml. Free HBGA stock was made from a mixture of PGM (1.0 mg/ml) and type B saliva at a 10:1 ratio. Ninety microliter aliquots of TV stock were incubated with either 10 μl free HBGA stock or PBS (as control) at 37°C for 1 h. One hundred microliters of each bacterial strain was added to the free HBGA-incubated TV stock aliquots, and incubated at 37°C and 50 rpm for 1 h. Bacteria were recovered from solution by centrifugation at 12,000 RCF for 1 min. The bacterial pellet was washed three times by vortex re-suspension with PBS and re-pelleted by centrifugation, and finally re-suspended into 140 μl of PBS. Viral RNA was extracted from bacteria-bound TV using a commercial viral RNA extraction kit (“QIAamp RNA Viral Mini,” Qiagen, Valencia, CA, United States) in accordance with the manufacturer’s protocol. Primers and probes were synthesized with modified fluorophores and quenchers (Integrated DNA Technologies, Inc.; San Diego, CA, United States). The primers and probes used for detection of TV were: TV forward (5′-TGA CGA TGA CCT TGC GTG-3′), TV reverse (5′-TGG GAT TCA ACC ATG ATA CAG TC-3′), TV probe (5′ HEX- ACC CCA AAG CCC CAG AGT TGA T -BHQ-1 3′). Extracted viral RNA was quantitated by probe-based quantitative real-time RT-PCR using a one-step RT-qPCR kit (“Quantitect Probe RT-PCR Kit,” Qiagen, Valencia, CA, United States) scaled to half-reaction volumes, but otherwise in accordance with the manufacturer’s protocol. Each 25 μl reaction consisted of 12.5 μl of Quantitect Probe RT-PCR master mix, 7.5 μl of RNAse-free water, 0.75 μl of each primer (TV forward, TV reverse, both at 10 μM), 0.25 μl of TV probe at 10 μM, 0.25 μl of Quantitect RT mix, and 3 μl of extracted RNA. Cycling times and temperatures were 50°C for 30 min (reverse transcription), 95°C for 15 min (denaturation of reverse transcriptase, and activation of Taq polymerase), followed by 45 cycles of 95°C for 15 s, 53°C for 20 s, and 60°C for 50 s (thermal cycling). Fluorescence was read at the end of each 60°C extension step. Automated thermal cycling and data acquisition was performed on a MX3000P qPCR system and MxPro software (Stratagene; La Jolla, CA, United States), with threshold determination at default settings. TV and RNase-free ddH_2_O were used as positive control and negative control, respectively. Unitless Ct values represented the viral genomic signal, which was converted to genomic copies by a recombinant-plasmid-based standard curve (Tian et al., 2013). The experiments in comparison with each other were done on the same plate with known amount TV viral RNA used as an internal standard.

### Partial and Full Heat-Inactivation for TV

TV stock was incubated with *E. coli* O86:H2, K-12, and PBS (as a control) at ratios of 1:1 and 100:1 at room temperature for 1 h. Three hundred microliter aliquots of TV-*E. coli* stock in 1.5-ml microcentrifuge tubes were heat-denatured at partial (56°C for 10 min) or full (56°C for 30 min) inactivation conditions, and quickly cooled in ice water bath. The control samples were kept at room temperature (Wang et al., 2014).

### Detection of Viral Infectivity by Culture-Mediated-Amplification RT-qPCR Assay (CMA-RT-qPCR)

CMA-RT-qPCR assay was used to determine infectivity of TV (Xu et al., 2015). Briefly, 24-well tissue culture plates (Corning Corporation, Corning, NY, United States) were seeded with LLC-MK2 cells in CMEM and incubated overnight to reach 80–90% confluency. CMEM was carefully removed from each well, 100 μl/well of TV stock, TV-bacteria complex, and heat-denatured versions of the prior was added, and incubated at 37°C for 1 h. Media was then carefully removed and residual virus-stock/bacterial-complex was removed from the wells by rinsing twice with MEM. Five hundred microliters of MEM was added to each well, and incubated at 37°C for 48 h. One hundred and forty microliters of putatively virus-enriched media was removed from each well and extracted for viral RNA using a commercial viral RNA extraction kit as described in a previous section. Viral RNA was quantitated for TV genomic signal using a commercial RT-qPCR kit as described in a previous section.

### Data Analysis and Statistics

Each experiment was repeated at least three times as independent replicates. All Ct values were converted to genomic copies. The data was then log-transformed and analyzed by one way analysis of variance. Detailed comparison was listed in legend section. *p* < 0.05 was considered significant.

## Results

### Type B HBGA-Like Molecules Expression Was Detected in *E. coli* O86:H2

**FIGURE 1:**
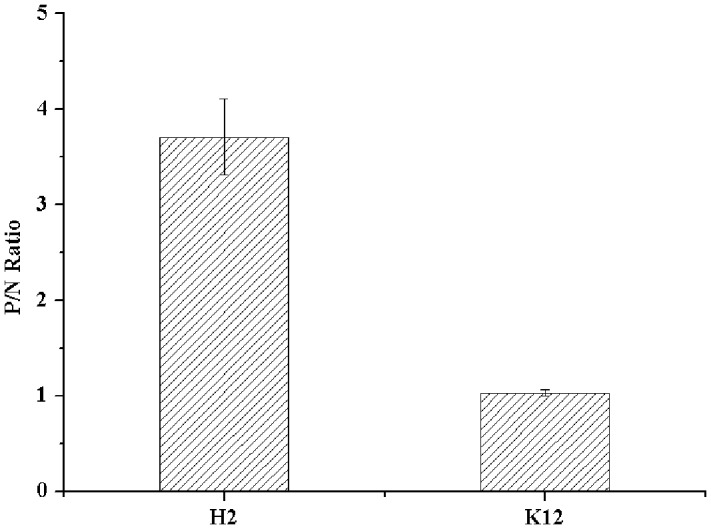
Detection of HBGA present in *Escherichia coli* O86:H2 measured by ELISA. H2: *E. coli* O86:H2; K12: *E. coli* K-12. The means and standard deviations from independent experiments were presented. Error bars represent standard deviation.

The expression of type B HBGA-like molecules in *E. coli* O86:H2 was confirmed by ELISA using a panel of MAbs against HBGA-like molecules. *E. coli* K-12 was used as a bacterial control for non-specific binding. The OD readings from wells omitted of primary antibodies (specific for TV) were used as N for calculation of P/N ratios. Only type B HBGA-like molecule was detected in *E. coli* O86:H2 with a P/N ratio of 3.71 (**Figure 1**). The P/N ratio for *E. coli* K-12 was 1.03. No other HBGA-like molecules were detected in *E. coli* O86:H2 and K-12 by using MAbs listed in Section “Materials and Methods” (data not shown).

### TV Bound to *E. coli* O86:H2 and K-12 Measured by ELISA

**FIGURE 2:**
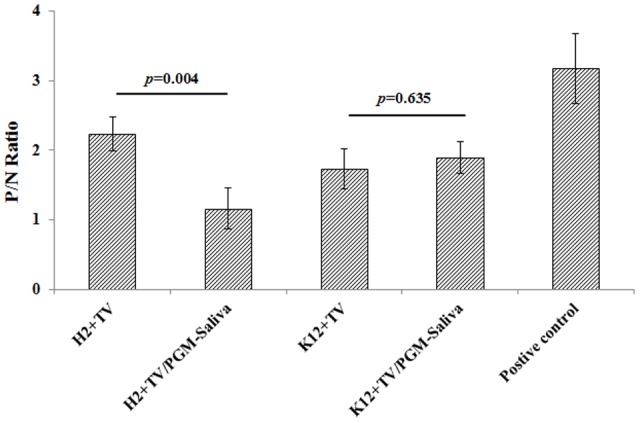
Binding of TV to *E. coli* O86:H2 and K-12 measured by ELISA. H2: *E. coli* O86:H2; K12: *E. coli* K-12; TV: Tulane virus; PGM: type III porcine gastric mucin; PGM-Saliva: PGM and blood type B saliva mixture (10:1). The means and standard deviations from independent experiments were presented. Error bars represent standard deviation. H2+TV/PGM-Saliva was compared with H2+TV; K12+TV/PGM-Saliva was compared with K12+TV by *t*-test. PGM-Saliva coated plate was used as a positive control for TV capture and detection.

Binding of TV to *E. coli* O86:H2 was detected by ELISA using TV-specific polyclonal antibodies. The P/N ratios for *E. coli* O86:H2 and K-12 were 2.23 and 1.90, respectively (**Figure 2**). Binding was further studied using a competition assay (**Figure 2**). The binding of TV to *E. coli* O86:H2 could be competitively inhibited by prior incubation of TV with free HBGA, reducing the P/N ratio from 2.23 to 1.16 (*p* = 0.004). The binding of TV to *E. coli* K-12 was not inhibited by prior incubation of TV with free HBGA. In contrast to *E. coli* O86:H2, the P/N ratio of K12 slightly increased from 1.89 to 2.03 after incubation of TV with free HBGA. Although P/N ratio of 2.0 was used to threshold for ELISA, it is very arbitrary. Therefore, RT-qPCR was used as additional approach to determine the binding of TV on *E. coli* O86:H2 and *E. coli* K12.

### TV Bound to *E. coli* O86:H2 and K-12 Measured by RT-qPCR Assay

**FIGURE 3:**
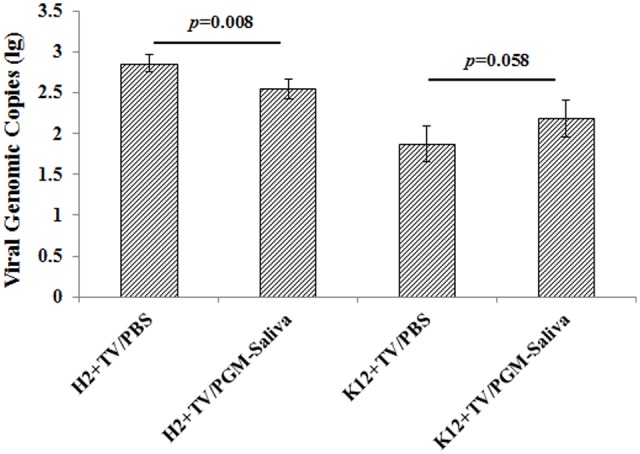
Binding of Tulane virus to *E. coli* O86:H2 and K-12 measured by RT-qPCR assay. H2: *E. coli* O86:H2; K12: *E. coli* K-12; TV: Tulane virus; PGM: type III porcine gastric mucin; PGM-Saliva: PGM and blood type B saliva mixture (10:1). The means and standard deviations from independent experiments were presented. Error bars represent standard deviation. H2+TV/PGM-Saliva was compared with H2+TV/PBS; K12+TV/PGM-Saliva was compared with K12+TV/PBS by *t*-test.

Binding of TV to both *E. coli* O86:H2 and K-12 was indicated by RT-qPCR. TV was able to bind to *E. coli* O86:H2 better than to *E. coli* K-12, with average Ct values of 28.21 and 31.45, respectively. Ct values converted to amplicon copies suggest that *E. coli* O86:H2 captures roughly five times as much TV as *E. coli* K-12 (**Figure 3**). Prior incubation with free HBGA reduced the ability of *E. coli* O86:H2 to capture TV by 57% (*p* = 0.008, *t*-test). In contrast, prior incubation with free HBGA did not reduce the ability of *E. coli* K-12 to capture TV. In fact, a numerical doubling enhancement of binding was observed (*p* = 0.058, *t*-test).

### Binding of Bacteria Does Not Protect Tulane Virus from Heat-Inactivation

**FIGURE 4:**
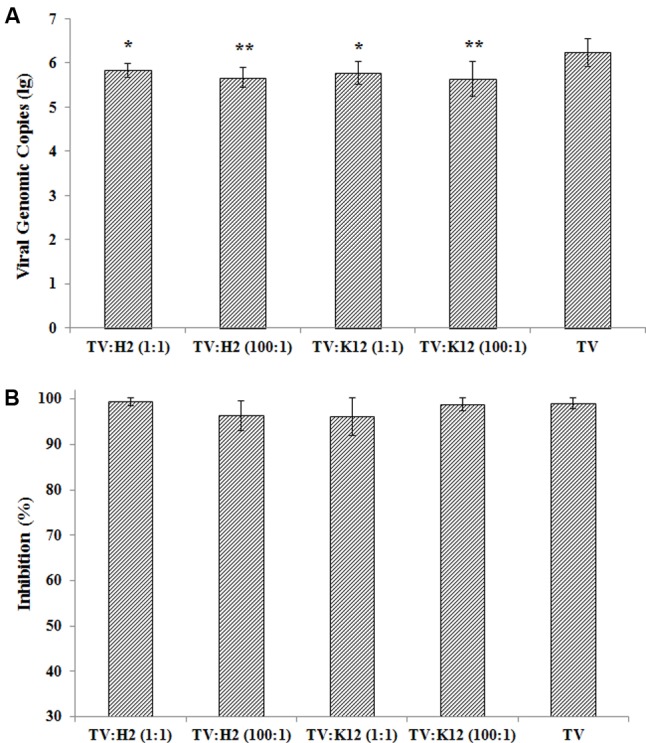
**(A)** TV replication in the presence of bacteria and absence of bacteria. H2: *E. coli* O86:H2; K12: *E. coli* K-12; TV: Tulane virus; ratio of TV to bacteria was listed in parenthesis. Error bars represent standard deviation. Groups of bacteria-virus-complex were compared with TV by one way analysis of variance (Brown–Forsythe method). ^∗^*p* < 0.05, ^∗∗^*p* < 0.01. **(B)** Inhibition of TV replication under heat-inactivation. The means and standard deviations from independent experiments were presented. Inhibition of viral replications after treatment of 56°C for 10 min calculated by the formula: 1-(viral genomic copy with treatment divided by viral genomic copy without treatment) %. One way analysis of variance (Kruskal–Wallis method) was used for statistical analysis of log-transformed data (*p* = 0.832) and Brown–Forsythe method was used to analysis % inhibition (*p* = 0.367). No significant difference among all groups tested in both methods.

TV was incubated with *E. coli* O86:H2 or K-12 at ratios of 1:1 and 100:1, and heat-denatured at partial (56°C for 10 min) and full (56°C for 30 min) inactivation conditions (**Figure 4**). The amplification of TV in LLC-MK2 cells was measured at 48 h post-infection by RT-qPCR assay. Incubation with bacteria reduced the attachment of TV to host cell culture, in-turn reducing its replication (**Figure 4A**). In the absence of bacteria, the TV titer was 1.72 × 10^6^ genomic copy after 48 h post-infection. The viral titers were 6.81 × 10^5^/ml (*p* = 0.012), 6.01 × 10^5^/ml (*p* = 0.010), 4.62 × 10^5^/ml (*p* = 0.002), and 4.32 × 10^5^/ml (*p* = 0.002) for TV w/O86:H2 at 1:1, TV w/K-12 at 1:1, TV w/O86:H2 at 100:1, and TV w/K-12 at 100:1, respectively (Holm-Sidak method). Binding to *E. coli* O86:H2 or K-12 did not seem to confer to TV any protection against heat-denaturation. There was no significant difference in viral titers in the presence of bacteria and absence of bacteria when TV was heat-inactivated (*p* = 0.83, Kruskal–Wallis one way analysis of variance on ranks). Heat-denaturation at partial inactivation conditions (56°C for 10 min) of TV and TV-bacteria complex produced viral genomic copies of 1.18 × 10^4^/ml, 2.10 × 10^3^/ml (*p* = 0.32), 1.46 × 10^4^/ml (*p* = 1.0), 7.89 × 10^3^/ml (*p* = 0.96), and 3.16 × 10^4^/ml (*p* = 0.81) for TV, TV w/O86:H2 at 1:1, TV w/K-12 at 1:1, TV w/O86:H2 at 100:1, and TV w/K-12 at 100:1, respectively (*t*-test, *p* > 0.05). Heat denaturation at partial inactivation conditions produced a similar reduction in viral replication regardless of the presence of bacterial binding, with loss percentages of 99.03, 99.42, 96.32, 96.10, 98.88% for TV, TV w/O86:H2 at 1:1, TV w/O86:H2 at 100:1, TV w/K-12 at 1:1, and in TV w/K-12 at 100:1, respectively (**Figure 4B**). There was no statistical difference among groups in the presence of bacteria and absence of bacteria (*p* = 0.367, Brown–Forsythe). Heat denaturation at full inactivation conditions (56°C for 30 min) produced total eradication of viral replication regardless of the presence of bacterial binding, and produced no detectable Ct values.

## Discussion

The interaction between HuNoVs and HBGAs has been demonstrated (Huang et al., 2003, 2005; Hutson et al., 2004). However, the role of HBGA in the viral replication cycle remains unclear. HBGAs have been considered putative viral receptor or co-receptors. HuNoV’s ability to bind to various HBGAs is an adaptation that might have played an important role in viral evolution (Singh et al., 2015a,b). HBGA-like moieties are present on the surface of some enteric bacteria (Springer et al., 1961; Yi et al., 2006; Miura et al., 2013; Li et al., 2015). Type H HBGA-like molecules found in *Enterobacter cloacae* could specifically bind HuNoV (Miura et al., 2013). Li et al. (2015) tested 11 bacterial strains for the presence of HBGA-like molecules and found expression in 8, with 5 strains capable of binding HuNoV rVLPs (recombinant VLPs). Li et al. (2015) further demonstrated that HuNoV rVLPs could be protected from acute heat stress by being bound to HBGA-like molecules-expressing bacteria (Li et al., 2015). This result was consistent with a previous report observing that cell attachment and environmental stability of poliovirus was enhanced by the binding of bacterial surface polysaccharides (Robinson et al., 2014). It needs to be noted that the Robinson study was conducted with viable poliovirus, while the Li study was conducted with VLPs rather than viable viruses. The measure of resistance to heat-stress was the persistence of the ability for VLPs or VLP-bacteria complex to bind to HBGA-coated wells after heat-denaturation. Therefore, there has been no direct evidence showing the HuNoVs would be protected against heat stress by the binding of HBGA-like molecules-expressing bacteria. In this study, we used TV as a surrogate to test the impact of HBGA-like molecules-expressing bacteria on viral inactivation by heat denaturation.

We demonstrated that type B HBGA-like molecules were expressed in *E. coli* O86:H2, but not in *E. coli* K-12 which shares a similar genetic background with O86:H2 except for HBGA-like molecules expression. We found that TV could bind to both *E. coli* O86:H2 and K-12, with O86:H2 manifesting more binding capacity. In addition, TV binding to intact bacterial cells was also suggested by RT-qPCR assay. The binding of TV to *E. coli* O86:H2 was partially HBGA-like molecules-mediated, as competitive binding from free HBGA could significantly reduce the quantifiable TV binding through both ELISA and RT-qPCR quantitation methods. In contrast, binding of TV to *E. coli* K-12 was nor reduced but slightly enhanced by competition from free HBGA. In conjunction with the observation through RT-qPCR that binding of TV to *E. coli* O86:H2 could not be completely inhibited by free HBGA, we believe we have observed the manifestation of both HBGA-like molecules-mediated and a yet-to-be-fully-characterized TV binding effects. Only this yet-to-be-fully-characterized TV binding was manifested by *E. coli* K-12. It is possible that molecules other than HBGA-like molecules present in both *E. coli* O86:H2 and K-12 are involved in the binding of TV. A recent report indicated that HuNoVs were able to bind to 10 bacteria strains with high efficiency (five Gram-positive and five Gram-negative strains) from human gut microbiota (Almand et al., 2017). GI and GII HuNoVs could bind to all 10 strains and TV could bind to 5 strains in this study. The viruses could be found in bacterial outer cell membrane, pili, or both by TEM (Almand et al., 2017). The interaction between these bacterium and HuNoVs or TV were considered specific as Turnip Crinkle virus did not bind to these bacterium (Almand et al., 2017). However, there was no evident that these tested bacterium all expressed HBGA-like molecules. We propose that HuNoV and TV could bind to molecules other than HBGA-like molecules. Murakami reported that binding of HuNoVs to human intestinal epithelial cells was independent of HBGA (Murakami et al., 2013). Tamura reported that GI VLPs could bind to a 105-kilodalton cellular binding protein (Tamura et al., 2000) and GII HuNoV could bind to heparan sulfate proteoglycan associated with the cellular membrane (Tamura et al., 2004). Gandhi et al. (2010) reported that GI VLPs could bind to lettuce by molecules other than HBGA. It has been reported that TV could use multiple receptors, including type A, type B and sialic acids (Tan et al., 2015; Zhang et al., 2015). Currently, we are in the process of identifying in bacteria participants other than HBGA-like molecules responsible for HuNoVs binding.

The infectivity of a culturable virus was traditionally determined by plaque assay and/or 50% tissue culture infectious dose (TCID_50_) assay, both of which are time-consuming and labor-intensive. It requires at least 5 days for optimal results. Recently, we developed the CMA-RT-qPCR as an alternative method to rapidly determine the infectivity of TV (Xu et al., 2015). With this assay, the input viral genomic signal could be detected at 6 h post-infection, became undetectable at 12 h post-infection, and the newly amplified viral genomic signal could be detectable at 24 h post-infection. Previously, we demonstrated that this method was comparable to TCID_50_ in determination of inactivation status of TV caused by various inactivation conditions including heat, chlorine, and UV-inactivation (Xu et al., 2015). Therefore, we used this method to determine the infectivity of the virus in this study.

The effect of the presence of bacteria on norovirus viral infectivity has never tested. It has been reported that the presence of enteric bacteria could enhance rotavirus binding/entry to host cells (Uchiyama et al., 2014). In this study, we demonstrated incubation with bacteria slightly reduced the attachment/replication of TV in LLC-MK2 cells. However, we found that binding of neither *E. coli* O86:H2 or K-12 conferred any protection to TV from inactivation by heat-denaturation. This finding is contrary to the implications of a prior report by Li et al. (2015). However, significant differences between these two studies may explain the varying conclusions, including the different strains of bacteria used in the studies, viral components, and targets for the end results. In the Li et al. (2015) study, HuNoV rVLPs were utilized instead of infectious viruses. As such, the only possible quantitation was that of binding (and loss-of-such) between rVLPs and HBGA-like molecules, but not the loss of infectivity. In this study, we quantitated viral replication as the indicator of viral infectivity, which in-turn is the indicator of resistance to heat-denaturation. We were able to achieve a more-direct demonstration that the binding of TV to bacteria regardless of HBGA-like molecules expression did not provide protection to the virus against heat-denaturation.

## Author Contributions

PT and QL designed the experiments. QL carried out the experiments with assistance from DW, DY, LS, and PT. PT and QL conducted statistical analysis. PT and QL wrote the paper. DY modified the paper. All authors reviewed the results, made substantial contributions and approved the final version of the manuscript.

## Conflict of Interest Statement

The authors declare that the research was conducted in the absence of any commercial or financial relationships that could be construed as a potential conflict of interest.
